# Selective Emotional Dysregulation in Splenium Agenesis. A Case Report of a Patient With Normal Cognitive Profile

**DOI:** 10.3389/fpsyg.2019.00631

**Published:** 2019-03-22

**Authors:** Sara Palermo, Agata Andò, Adriana Salatino, Stefano Sirgiovanni, Luana De Faveri, Antonella Carassa, Maria C. Valentini, Rosalba Morese

**Affiliations:** ^1^Department of Psychology, University of Turin, Turin, Italy; ^2^Neuroradiology Division, Azienda Ospedaliera-Universitaria Città della Salute e della Scienza di Torino, Turin, Italy; ^3^Faculty of Communication Sciences, Università della Svizzera Italiana, Lugano, Switzerland

**Keywords:** MRI, splenium of the corpus callosum, emotional dysregulation, neuropsychological tests, social development

## Abstract

**Objective:** Patients with lesions of the corpus callosum are rare and may present different symptoms of the disconnection syndrome. However, to-date studies on callosotomized patients have not been conclusive, likely because of the non-uniform nature of clinical features, the extent of resection, and methods used to investigate specific and related deficits. Agenesis of the corpus callosum (AgCC) may be asymptomatic and discovered incidentally or associated with very slight deficits diagnosed during neurological examinations. In this study, we reported a case of an apparently completely asymptomatic 23-year-old woman with appreciable agenesis of the splenium of the corpus callosum.

**Methods:** She underwent a neurological evaluation, a comprehensive battery of neuropsychological tests to identify any subclinical dysfunction that may affect the functionality of the subject in the daily life. Specifically, the possible presence of emotion dysregulation was examined by using a self-report questionnaire.

**Results:** She showed normal neuropsychological and emotional functioning, performing efficiently on tests measuring acquired brain impairment.

**Discussion:** The present case is discussed in terms of neuroplasticity – with a focus on putative compensatory mechanisms – emphasizing the variegated clinical feature patterns of brain defects present from birth.

## Introduction

The corpus callosum is the largest white matter commissure connecting the cerebral hemispheres ( Aboitiz and Montiel, 2003; Schell-Apacik et al., 2008). It is regarded as having a role of transfer, integration and coordination of information between homologous brain areas and as being involved in learning, memory, thinking, three-dimensional visual ability, executive functions, as well as visual reaction time ( Huang et al., 2015).

Agenesis of the corpus callosum (AgCC) is estimated to occur in approximately 0.2–0.7% of the general population ( Schell-Apacik et al., 2008; Sefidbakht et al., 2016). Its prevalence in children with developmental disorders is as high as 230 in 10,000 ( Jeret et al., 1986). AgCC encompasses complete absence as well as hypogenesis of the corpus callosum ( Paul et al., 2007); the cause of AgCC in humans is not yet sufficiently known (only in 30–45% of cases). In the remaining 55–70% of individuals with AgCC, callosal agenesis is often an incidental neurological finding ( Paul et al., 2014).

AgCC is associated with a wide range of cognitive, behavioral and neurological deficits ( Schilmoeller and Schilmoeller, 2000). Three “cognitive patterns” observed in individuals with AgCC are the following: (1) neurodevelopmental disorders (i.e., autism spectrum); (2) normal intellectual ability (despite the presence of subtle neuropsychological deficits/abnormalities in the social cognition domain); and (3) overt neuropsychological impairment ( Chiappedi and Bejor, 2010). AgCC is most often associated with mild limitations in tasks that involve interhemispheric transfer ( Lassonde et al., 1991).

Slightly fewer than 33% of individuals with AgCC have “normal” or only slightly delayed development ( Shevell, 2002) while the prognosis ranges from severely delayed to perfectly normal ( Paul et al., 2007). For example, Lum et al. (2011) reported fMRI findings in three cases of asymptomatic AgCC. Nevertheless, when sensitive standardized neuropsychological measures are applied, a certain degree of impairment in higher-order cognition or social skills is observed ( Paul et al., 2007). For example, AgCC individuals’ cognitive ratings of valence and arousal may show greater variance and may be less sensitive to negatively valenced stimuli ( Paul et al., 2007). Moreover, AgCC subjects have been found to exhibit social-cognitive deficits and poor understanding of social-emotional aspects similar to those found in autism ( Paul et al., 2004; Barnes et al., 2009; Turk et al., 2010; Lombardo et al., 2012). Interestingly, AgCC subjects can show impaired awareness of social and cognitive abilities ( Brown and Paul, 2000; Paul et al., 2007), leading to delays in cognitive-behavioral profiling and difficulties in therapeutic engagement.

A few studies have been concerned the partial AgCC, in particular regarding the splenium. Knyazeva (2013) reported that ≪*in the adult human brain, the function of the splenium in a given area is defined by the specialization of the area and implemented via excitation and/or suppression of the contralateral homotopic and heterotopic areas at the same or different level of visual hierarchy*≫ (page 1). The anterior part contains thin late-myelinating fibers connecting the parietal and medial temporal regions, whereas the thick early-myelinating fibers connect primary/secondary visual areas ( Barkovich and Kjos, 1988; Sano et al., 2008). The myelination pattern likely reflects the underlying complex splenium microarchitecture ( Whitehead et al., 2014). The associative cortex actually develops after the primary cortical areas ( Gogtay et al., 2004).

Knyazeva (2013) reported that ≪*splenial fibers across brain areas are involved in a variety of functions, while their considerable variation between subjects implies a contribution of the splenium to plastic changes in the course of human development*≫ (page 2).

The above considerations have led to specific questions about the long-term consequences of partial AgCC and whether lightweight behavioral-emotional symptoms might be able to escape detection due to coverage phenomena.

The present case report describes a young woman with agenesis of the splenium of the corpus callosum who appeared to be asymptomatic for her entire life.

At the time, we hypothesized that despite the apparent absence of symptoms, slight neuropsychological abnormalities could be hidden in the patient’s life history. In particular, we believed that behavioral abnormalities – of such entity as not to affect the patient’s daily life - could have emerged to a more careful evaluation of components generally not considered according to a purely neurological approach.

## Materials and Methods

A 23-year-old woman was admitted to the Neuroradiology Division to participate in an experimental fMRI study as part of the “normal” control group. Inclusion criteria were the following: (1) no past or current mental illness assessed using the Mini-International Neuropsychiatric Interview (MINI) Plus: in particular, no major depression, dysthymia, mania or disinhibition based on DSM-V criteria; (2) no family history of mental illness or psychiatric hospitalization; (3) no history of head injury with loss of consciousness; (4) a Mini Mental State Examination (MMSE) ( Folstein et al., 1975) score of ≥27; (5) no current pharmacological treatment, substance abuse or dependence that could substantially affect cognitive functioning.

At the pre-scan evaluation, the young woman was alert, conscious and oriented in time and space. Production and comprehension of language were preserved. The neurological examination was negative. The psychological interview for inclusion in the normal control group highlighted normal mental status and psychological adjustment, intellectual and problem-solving ability, processing speed and executive functioning.

The young woman^1^ had a normal postnatal developmental history and complete functional independence of long-living. She has reached the main milestones of young adulthood. Moreover, she denied any functional or social dysfunction in everyday life.

### Neuroimaging

**FIGURE 1:**
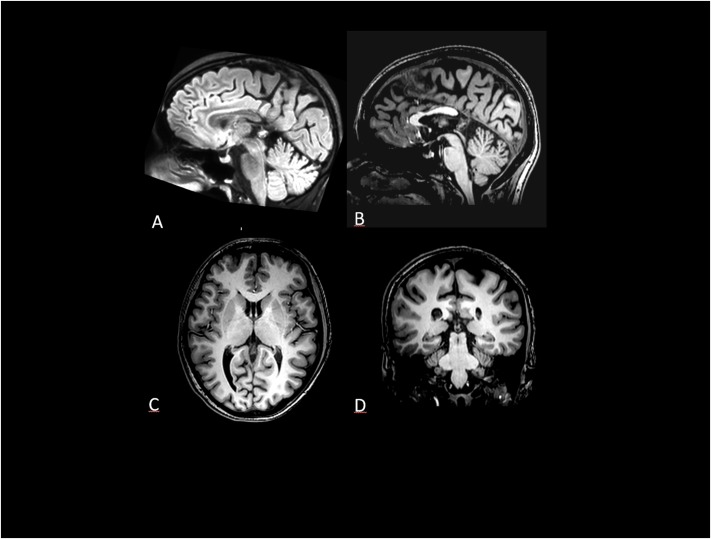
**(A)** Sagittal FLAIR 3D volume rendering and **(B)** Sagittal T1-WI shows normal rostrum, genu and body of corpus callosum and absent splenium (arrow) **(C)** Axial section through cavum septi pellucidi and internal cerebral vein within the cistern of the velum interpositum. **(D)** Coronal T1-WI: the retrothalamic cistern and medial wall of the lateral ventricles atri are delimited by cingulis and precuneus gyrus due to the splenium absence.

Neuroimaging data was acquired using a 3T Philips Ingenia scanner. Structural images were collected using T1-weighted (TR = 8.1 ms, TI = 900 ms, TE = 3.7 ms, voxel size = 1 × 1 × 1 mm^3^) and axial T2-weighted FSE (TR = 3200 ms, TE = 100 ms, FOV = 432 × 432 mm, section thickness = 4 mm, acceleration factor = 2) sequences. The MRI showed no alterations of the brain parenchyma in the supra and infratentorial sites. Ventricles and sulci were of normal size and aligned. However, unexpected appreciable agenesis of the splenium of the corpus callosum was found ( Figure 1) with no alterations in the brain parenchyma signal in the supra and infratentorial sites.

In view of such neuroimaging findings, YW was referred to the Neuroradiology Division in order to undergo a thorough neuropsychological evaluation ( Palermo et al., 2015). The neurologist conducted a standardized interview with the patient on her health history before formalizing the diagnostic question to the neuropsychologist. The format of the health history is structured in order to organize patient information by following a written report for other health professionals. Subsequently, the neuropsychologist carried out a psychological anamnesis: it has the purpose of investigating aspects that serve to provide useful indications for the more possible precise psychological diagnosis. The complete interview is divided into the following topics: demographic data and marital status; physiological anamnesis; recent, near and remote anamnesis; functional anamnesis; psychological and psychopathological anamnesis; family history.

### Neuropsychiatric Assessment

The *Brief Psychiatric Rating Scale 4.0* (BPRS 4.0) was used to measure neuropsychiatric symptomatology ( Roncone et al., 1999). Specific scales were employed to assess behavioral mood changes: the *Apathy Evaluation Scale-Clinician version* (AES-C) ( Marin et al., 1991); the *Hamilton Depression Rating Scale* (HDR-S) ( Hamilton, 1969); the *Young Mania Rating Scale* (YMRS) ( Young et al., 1978). The *Self-Regulation Skills Interview* (SRSI) ( Ownsworth et al., 2000, 2002), a clinical tool developed to measure higher levels of self-awareness and self-regulation skills that imply insight, motivation and compensation was also administered ( Palermo et al., 2014).

The *Toronto Alexithymia Scale* (TAS-20) ( Bagby et al., 1994) is one of the most widely used tools for measuring alexithymia. Alexithymia refers to individuals who are unable to identify and describe their emotions or have a tendency to minimize their emotional experience and to focus their attention externally. Items are rated on a 5-point Likert scale. The TAS-20 uses cut-off scoring: ≤51 = non-alexithymia, ≥61 = alexithymia.

The *Difficulties in Emotion Regulation Scale* (DERS) ( Gratz and Roemer, 2004) was administered to assess multiple aspects of emotion dysregulation. DERS is a 36-item, self-report questionnaire comprising six subscales developed to detect multiple aspects of emotion dysregulation: (1) *Non-acceptance of emotional responses* (Non-acceptance); (2) *Difficulties engaging in goal directed behavior* (Goals); (3) *Impulse control difficulties* (Impulse); (4) *Lack of emotional awareness* (Awareness); (5) *Limited access to emotion regulation strategies* (Strategies); (6) *Lack of emotional clarity* (Clarity) ( Gratz and Roemer, 2004). Recently, Giromini et al. (2017) provided equations to calculate age- and gender-adjusted T-scores, so that clinicians would easily interpret the resultant T-transformed DERS scores, which have a mean of 50 and standard deviation of 10. DERS scores between 65 and 70 T should indicate the presence of difficulties in emotion regulation, whereas scores higher than 70 T indicate important problems in emotion regulation. DERS reported high alpha coefficients ranging from 0.80 to 0.89 for the different scales. Exploratory factor analysis was used to provide data on the factor structure of the DERS and identify the underlying dimensions of emotion regulation as assessed by this tool. We obtained T-transformed DERS scores. As mentioned before, DERS scores between 65 and 70 T indicate the presence of problems in emotion regulation and scores higher than 70 T indicate significant problems in emotion regulation.

### Neuropsychological Assessment

Intelligence was assessed using the *Wechsler Adult Intelligence Scale – IV edition* (WAIS-IV) ( Lang et al., 2015). The pre-morbid intelligence quotient was estimated using the *Brief Intelligence Test* (TIB) ( Sartori et al., 1995; Colombo et al., 2002) that is the Italian version of Nelson’s National Adult Reading Test ( Nelson, 1982). Cognitive abilities were analyzed through a comprehensive battery of tests to measure ( Palermo et al., 2018a): attention through *Attentional Matrices* ( Spinnler and Tognoni, 1987); executive functions by means of *Trial Making Test* parts A, B ( Reitan and Wolfson, 1994); memory through *Wechsler Memory Scale -Subtests 4 and 7* ( Wechsler, 1945); language oral production by means of *verbal fluency* ( Spinnler and Tognoni, 1987). The absence of unilateral visual-attentional neglect was assessed by menas of the *Bells Test* ( Gauthier et al., 1989). Dysexecutive syndrome was assessed using ecological neuropsychological tasks created to simulate daily life situations: the *Behavioral Assessment of the Dysexecutive Syndrome* test battery (BADS) ( Wilson et al., 1996). Theory of Mind visual stories (ToM1 and ToM2) were used to test perspective-taking skills ( Amanzio et al., 2008), while the *Reading the Mind in the Eyes* task (RME) was used to measure social cognition and emotion recognition abilities ( Baron-Cohen et al., 2001). Metacognition was assessed with the metacognitive version of the *Wisconsin Card Sorting Test* (m-WCST) ( Koren et al., 2006).

## Results

Young women had a normal cognitive and intellectual level and obtained normative scores on all the neuropsychological tasks that were administered. The IQ total score – determined by means of the WAIS-IV – was 108 (average range). Not only visual scanning and visual integrative skills were within the normal range at the clinical observation, but visuospatial abilities were preserved when evaluated through WAIS-IV sub-tests. Visuo-constructional ability on the Block Design subtest was above average. Scores for the accessible fund of knowledge, concept information, knowledge of word meaning, verbal fluency, and word reading were all within the normal range. Abstract reasoning was within the normal range. Her ability to generate and test concepts when presented with a novel problem-solving task was normal. Immediate and delayed recall for verbal narrative were normal. Behavioral programs initiation was within the normal range. Moreover, YW did not show difficulties at cognitive level. In addition, attention and mental flexibility were also in line with normal limits. There was no dysexecutive syndrome as measured by the BADS.

From a neuropsychiatric point of view, test behavior was devoid of any impersistence, intrusions or perseveration. Scores of the neuropsychological evaluation are presented in Table 1.

**Table 1:** Neuropsychiatric and neuropsychological evaluation synopsis.

	Max score	YW’s scores	Cut-off
MMSE	30	30	≥23.8
Attentional matrices	60	52	≥31
TMT A	500	38	≤94
TMT B	500	64	≤283
TMT B-A		26	≤187
Bell test	35	35	≥32
Verbal fluency_ semantic		22.25	≥7.25
Verbal fluency_ phonetic		36	≥17.35
Wechsler memory_4	22	10	/
Wechsler memory_7	2, 5	19,5	/
TOM_1	4	4	≥3
TOM_2	4	4	≥3
RME	36	21	≥21
TIB_IQ TOT		112.15	90–110
TIB_IQ verbal		109.23	90–110
TIB_IQ performance		103.75	90–110
WAIS-R IQ_TOT		108	90–109
Bads total score	24	13	≥13
WCST %	100	75	≥37.1
WCST_errors %	100	25	/
WCST_pers errors %	100	31.25	≤42.7
Confidence	100	68.28	/
Accuracy	100	60	/
Free choice improvement		0.0125	/
Global monitoring		-0.7375	/
Monitoring resolution		-12	/
Control sensitivity		0.4375	/
Monetary gain		5.2	/
BPRS	168	30	/
YMRS	60	5	≤12
HDR-S	67	6	≤7
AES-C	72	56	≥37.5
SRSI	60	15	/
TAS-20	100	43	≤51


*Wherever there is a maximum score and/or a normative value, the cut-off scores are given in the statistical normal direction; the values refer to the normative data for healthy controls matched for age and education. Empty cell in the last column indicate the absence of a normative cut-off for that assessment tool; where there is no a normative cut-off, clinical practice and scale direction have been used to evaluate the subject’s performance. No abnormal value was detected by the neuropsychological assessment*.

The neuropsychiatric examination was almost normal, with the young woman’s scores on the YMRS, HDR-S, and AES-C all being below the cut-off point. Her SRSI scores appeared to indicate preserved insight. Moreover, alexithymia was not present as measured by the TAS-20.

The DERS scale scores indicate the overall tendency to accept negative emotions, knowledge of emotions, abilities to control impulsive behaviors and to use of situationally appropriate strategies to modulate own emotional responses (see Table 2). In addition, it is noteworthy that YW obtained low DERS scores (i.e., lower than 50 T) on the Goals and Clarity scales, which is referred to the ability to perform a goal-directed behavior and clarity of emotions (see Table 2).

**Table 2:** DERS‘s results.

	Scores	
	Raw	T
**DERS**		
Non-acceptance	14	52
Goals	8	35
Impulse	12	49
Awareness	7	35
Strategies	10	38
Clarity	5	35
Total	56	36


## Discussion

Patients with callosal alterations of different locations and extent or splenium agenesis should present distinct symptoms of disconnection syndrome ( Campbell et al., 1981). However, studies in patients who have undergone callosotomy have not been conclusive, likely because of the heterogeneity of patients’ clinical features and age, the extent of resection, and the different methods used to investigate the related deficits ( Bentin et al., 1984; Badan and Caramazza, 1997). While AgCC may be associated with neurological problems, it is thought that such conditions are due to anomalies in cerebral connections rather than in the corpus callosum itself ( Sande and Garge, 2010). Considering the splenium of the corpus callosum, Park et al. (2014) reported that not only individuals who only had *in situ* lesions displayed relatively mild symptoms, but also they had no impaired cognition (unlike when multiple lesions in other callosal areas were ascertained).

In this case report, the fact that YW’s postnatal development history, long-term functional independence in her daily living, and current cognitive performance were normal supports the view that partial AgCC has not prevented normal cognitive development – unlike what happens in cases of complete agenesis ( Paul et al., 2007).

The young woman’s neuropsychological functions were preserved, and she performed well on tests capable of detecting brain dysfunction such as the BADS, WCST, and RME. Although scores on self-report scales in adults with primary AgCC often imply diminished self-awareness ( Brown and Paul, 2000), YW’s awareness appeared to be normal. The absence of two factors that might possibly contribute to low self-awareness, namely a generally impaired ability to understand or describe social situations ( Paul et al., 2007) and metacognitive-executive dysfunctions ( Palermo et al., 2014, 2015, 2017, 2018b; Morese et al., 2018), is an indication that the YW’s self-perception remained intact.

Although disconnection symptoms have been reported in AgCC, they were not exhibited by YM. Importantly, a disconnection syndrome is less likely to be exhibited by adults with AgCC and individuals who have undergone callosotomy during their childhood ( Barr and Corballis, 2002).

The neuropsychological diagnostic investigations have therefore confirmed what we previously hypothesized and substantiated by YM’s close and remote anamnesis: no obvious functional or cognitive impairment affects YM’s daily living.

How the absence of neuropsychological symptomatology could be explained – considering the presence of a cerebral structural anomaly that would suppose a functional repercussion?

A mechanism of functional compensation in AgCC has been identified in the hypertrophy of the anterior commissure ( Fischer et al., 1992). The authors described a case of a patient with AgCC and enlarged anterior commissure verified by magnetic resonance imaging (MRI) exams that have obtained normal scores on both visual and tactile interhemispheric transfer tasks. Our clinical case could suggest considerations for the idea that adults with AgCC might benefit from some form of neural compensation during the early stages of growth and development ( Paul, 2011).

The far-reaching plastic changes observed in AgCC and young patients who have undergone callosotomy might occur during a critical developmental phase that coincides with synaptic overproduction and redundancy ( Lassonde and Jeeves, 1991). Neural functioning in isolated AgCC is evidence of the far-reaching plastic changes that take place in the developing human brain ( Paul et al., 2014). Since resting-state functional brain connectivity can be surprisingly intact in isolated AgCC, it is possible that the brains of adults with AgCC might generate a typical, bilateral set of resting-state functional brain networks ( Tyszka et al., 2011). Tyszka et al. (2011) reported that ≪*almost all of the group-level independent components identified in controls were observed in AgCC and were predominantly bilaterally symmetric*≫ (page 15154). According to the authors, when the corpus callosum is absent, ≪*functional networks emerge flexibly with the development of normal cognition and behavior and can be realized in multiple structural architectures*≫ (page 15154).

Despite this evidence, interhemispheric functional connectivity in AgCC appears to vary more when patients are engaged in cognitive tasks ( Paul et al., 2014). Indeed, AgCC subjects do not have the features of the classic disconnection syndrome observed in patients who have undergone corpus callosotomy, but they might show mild disruption of interhemispheric transfer ( Paul et al., 2014). The limits of such compensation might become more apparent when performing tasks that involve complex cognitive operations, require fast processing, and are less reliant on previous experience ( Paul et al., 2007, 2014).

In line with these scientific findings, Hinkley et al. (2012) reported that disrupted callosal development can lead to reduced functional connectivity (FC) resulting in impairments in specific cognitive domains. Interestingly, the authors performed a planned comparison between patients with partial AgCC (pAgCC) and complete AgCC (cAgCC) in order to understand how callosal integrity can be associated with cognitive abilities. They concluded that concomitant anatomical tracts developed specifically in AgCC patients ( Tovar-Moll et al., 2007; Wahl et al., 2009) might affect inter- and intra-hemispheric connectivity and the related underlying cognitive mechanisms ( Hinkley et al., 2012). Indeed, a possible peculiarity in YW’s FC might therefore explain not only her normal cognitive profile but also her sufficient ability in accepting negative emotions, capacities to perform goal-directed behavior (this is the Goal scale), and clarity of her experienced emotions as reported by the Clarity scale. In fact, the scores YW obtained on all DERS scales are within the normal range (i.e., scores ≤50 T). She showed a good capacity of emotion regulation, although the Non-acceptance scale reported the highest score (*T* = 52) when compared to the scores obtained by the other DERS scales. This finding – as isolated and selective – substantiates what was our initial hypothesis: not at the cognitive level but at the level of subclinical behavioral-psychological slight impairment were to be looked for any deficit in the case of YW. In this regard, we may speculate that she usually tries to manage those negative emotions experienced as invasive by using rationalization as a defense mechanism in which negative feelings and emotions are explained in a seemingly rational manner to make them consciously tolerable (see the scores on the other DERS scales). Although previous studies have investigated the processing of verbal/psychophysiological indices of emotional arousal in relation to the left and right hemispheres ( Wahl et al., 2009; Morese et al., 2016; Giromini et al., 2016), the way the two hemispheres normally interact in order to produce emotional responses to stimuli is yet to be explained ( Paul et al., 2007). Some patients with AgCC reported high skin conductivity; in general, the level of skin-conductance responses can discriminate emotion between different categories ( Paul et al., 2007). The authors suggested that largely intact right hemisphere mechanisms might support psychophysiological emotional responses. This mechanism might cause YW to behave consistently with the socio-relational stimuli coming from the environment. In addition, Paul et al. (2006) also suggested that the absence of interhemispheric communication in AgCC may affect normal verbal ratings of arousal, a mechanism that is consistent with some alexithymia models. Indeed, a hallmark of alexithymia is the difficulty of putting emotional states into words ( Franz et al., 2008). Alexithymia is considered as a personality trait that is normally distributed in the population and is associated with increased risk of psychopathology ( Franz et al., 2008). Alexithymia is associated with an inability to regulate affect using adaptive processes like expressing or suppressing emotions, using imagination, modulating arousal, getting and making use of social support, coping with distress, cognitive assimilation, and accommodation ( Taylor et al., 1997). It is therefore thought to be among the factors contributing to conditions such as depression, anxiety, compulsive and addictive behavior, heightened or prolonged physiological arousal, physical symptoms, and even somatic symptom disorders ( Taylor et al., 1997). The fact that YW obtained a normative score on the TAS-20 scale supports the idea that compensatory brain plasticity mechanisms are intensified during development, which is why she is able to behave normally in daily life.

It has been postulated that the posterior midline regions may have an important role for self-referential and social-cognitive development. Thus, the case of YW, who had pAgCC, is possibly less extreme than it would have been with cAgCC ( Pfeifer and Peake, 2012). This aspect will be addressed in further clinical studies of our patient. Importantly, an approach that takes into account multiple levels of analysis, such as neuropsychological measures neuropsychological tests associated with functional neuroimaging, is needed in order to gain a clear understanding of YW’s behavioral and cognitive profile.

## Conclusion

AgCC can be entirely asymptomatic or associated with very slight deficits diagnosed during neurological examinations. With an incidence of just 1 in 4000 in the general population, AgCC is rare and cases are very difficult to find. Nevertheless, we believe that this case report sheds light on how a structural defect could have a more complex effect than the one already known about brain function and highlights the need for clear guidelines to manage incidental MRI findings in the research setting. Indeed, the present case report wants to be a driving force for a more careful evaluation of occasional MRI findings so that subclinical psychological alterations that might negatively affect the quality of life of patients can be recognized and treated where necessary. Given that distinct methods engage and assess distinct mental processes ( Bornstein, 2017), it is noteworthy that we used a multimethod assessment including different measures, in order to examine thoroughly the psychological functioning (resources and limits).

We suggested that theoretical models of brain alteration might be more effective if they integrate MRI, psychological and neuropsychological data, adopting a neurocognitive approach. In particular, a multidimensional approach involving instruments for the evaluation of complex, highly selective functions would be useful.

It would be important to replicate these observations on a group of patients, as the proposed conclusions could not be generalized. An important limitation of this work is undoubtedly the impossibility of assessing further cognitive domains in depth. This is because YW has been sent to our observation with an unspecific question by the attending neurologist. It would have been opportune to evaluate with greater attention the visuo-spatial abilities and the possible presence of spatial neglect. However, these elements seem to be not predictors of any anomalies in the DERS scores.

## Ethics Statement

The study was approved by the Ethics Committee “A.O.U. Città della Salute e della Scienza di Torino – A.O. Ordine Mauriziano – A.S.L. Città di Torino” as part of the core research criteria followed by the Neuroradiological Division. All the implemented procedures ensured the safety, integrity, and privacy of patients. Any critical aspects, neither with regard to the fMRI acquisition, nor to the neuropsychological assessment could be noticed. Importantly, the study has been conducted according to the principles set forth by the Declaration of Helsinki (59th WMA General Assembly, Seoul, October 2008) and in accordance with the Medical Research Involving Human Subjects Act (WMO). Written informed consent was obtained from YW both for the purposes of research participation as well as for the publication of the case report, including indirectly identifiable data.

## Author Contributions

The case-report study is based on a concept developed by RM who wrote the manuscript and took part in the review and critique processes as PI. Moreover, RM conducted the MRI execution. SP organized the study, performed the neuropsychological assessment (organization and execution), wrote the manuscript and participated in the review and critique processes. AA participated in the administration, scoring evaluation and interpretation of all the psychological results. Moreover, she participated in writing the manuscript and in the review process. SS and LDF conducted the MRI acquisition. MV organized and conducted the MRI acquisition, participated in the interpretation of results and in writing the manuscript. AS participated in writing the manuscript. AC participated in writing the manuscript (review and critique). All the contributors gave their approval of this version of the manuscript to be submitted.

## Conflict of Interest Statement

The authors declare that the research was conducted in the absence of any commercial or financial relationships that could be construed as a potential conflict of interest.

## Footnotes

1 By convention and to safeguard privacy, from this point forward the young woman will be indicated with the initials YW.
